# The insulin resistance-systemic vascular resistance-isolated diastolic hypertension axis: a metabolic framework for an overlooked hypertension phenotype

**DOI:** 10.3389/fcvm.2026.1834237

**Published:** 2026-06-05

**Authors:** Jiao-Yang Zhao, Yan Shu, Si-Hui Wang, Hong Wu

**Affiliations:** 1 The Second Clinical Medical College, Henan University of Chinese Medicine, Zhengzhou, China; 2 School of Pharmacy, Henan University of Chinese Medicine, Zhengzhou, China

**Keywords:** ambulatory blood pressure monitoring, endothelial dysfunction, insulin resistance, isolated diastolic hypertension, metabolic score for insulin resistance, sympathetic nervous system, systemic vascular resistance, triglyceride-glucose index

## Abstract

Isolated diastolic hypertension (IDH) is defined as elevated diastolic blood pressure (DBP) with systolic blood pressure (SBP) below guideline-specific thresholds for hypertension. IDH is more prevalent in young and middle-aged adults and generally declines with age, yet its prognostic significance and optimal management remain debated. Hemodynamically, IDH differs from isolated systolic hypertension in that it is typically characterized by increased systemic vascular resistance (SVR) with relatively preserved large-artery compliance, consistent with a resistance-vessel–dominant phenotype. Accumulating evidence links insulin resistance (IR) and compensatory hyperinsulinemia to mechanisms that promote SVR elevation. In this review, we propose an insulin resistance–systemic vascular resistance–IDH (IR–SVR–IDH) axis as a mechanistic and clinically actionable framework for a metabolically enriched subtype of IDH. We synthesize evidence across three interacting pathways: (i) neural mechanisms involving sympathetic activation and downstream renin–angiotensin–aldosterone system signaling; (ii) vascular mechanisms characterized by endothelial insulin resistance, impaired nitric oxide bioavailability, inflammation, and microvascular remodeling; and (iii) renal mechanisms related to preserved sodium-retaining effects of insulin, salt sensitivity, and volume expansion. Clinically, pragmatic IR surrogates—including the homeostasis model assessment of insulin resistance (HOMA-IR), triglyceride–glucose (TyG) index, and metabolic score for insulin resistance (METS-IR)—are associated with IDH risk and may facilitate metabolic enrichment and risk stratification. Emerging outcome data indicate that metabolic status modifies IDH-associated cardiovascular risk, underscoring the importance of integrated blood pressure and metabolic phenotyping. By integrating hemodynamic and metabolic characterization, this framework supports a precision-oriented approach to an often overlooked hypertension phenotype.

## 1 Introduction

Hypertension remains one of the most important modifiable risk factors for cardiovascular disease and premature mortality worldwide. Although clinical attention has traditionally emphasized systolic blood pressure (SBP), the clinical meaning of diastolic blood pressure (DBP)—and its value for risk stratification in specific populations—warrants reappraisal. Isolated diastolic hypertension (IDH) is defined as elevated DBP with SBP below the systolic threshold used to define hypertension, and diagnostic thresholds differ across guidelines. For example, the 2017 ACC/AHA guideline defines stage 1 hypertension as ≥130/80 mmHg, which operationally yields an IDH phenotype of DBP ≥ 80 mmHg with SBP < 130 mmHg; by contrast, the 2018 ESC/ESH guideline retains 140/90 mmHg as the office threshold for hypertension, corresponding to an IDH phenotype of DBP ≥ 90 mmHg with SBP < 140 mmHg (1, 2) Table 1). Using these definitions, IDH is more frequently observed in young and middle-aged adults, with prevalence generally declining with age (3–5).

**Table 1:** Definitions and phenotyping considerations for isolated diastolic hypertension (IDH).

Source	SBP criterion (office)	DBP criterion (office)	Out-of-office BP (ABPM/HBPM)	What this definition tends to capture	Pitfalls/boundaries
ACC/AHA 2017 (guideline BP categories)	Stage-based	Stage-based	Recommends out-of-office BP to confirm diagnosis	Lowers DBP threshold (80–89 as Stage 1) → expands early diastolic elevation pool	IDH often operationalized in studies; risk varies by threshold/population
ACC/AHA-style operational definition used in studies	SBP < 130	DBP 80–89	Prefer ABPM/HBPM confirmation when feasible	Stage 1 IDH phenotype under 2017 classification	Sensitive to office misclassification; ABPM-confirmed persistent IDH likely more informative
ESC/ESH 2018	SBP < 140	DBP ≥ 90	Out-of-office BP encouraged	Traditional IDH definition; typically higher DBP burden	Limited comparability with ACC/AHA-style IDH
ISH 2020 Global	SBP < 140	DBP ≥ 90	Recommends HBPM/ABPM for confirmation where possible	Practical global standard aligned with 140/90 office threshold	Method-specific thresholds; misclassification without ABPM/HBPM
ESH 2021 measurement practice	–	–	Strongly supports ABPM/HBPM for accurate phenotyping	Focuses on measurement/confirmation (white-coat, masked, nocturnal BP)	Not a phenotype-definition document; complements other thresholds
ESC 2024	SBP < 140	DBP ≥ 90 (HTN)	Recommends out-of-office BP for diagnosis	Adds elevated BP category; reinforces out-of-office confirmation	Transition from 2018 ESC/ESH may affect framing of borderline DBP

(1) IDH prevalence/risk estimates depend on threshold and measurement setting; this review emphasizes ABPM/HBPM-confirmed persistent IDH for clinical implications and trial design. (2) SBP/DBP criteria shown are office BP unless otherwise specified. ABPM, ambulatory blood pressure monitoring; ACC/AHA, American College of Cardiology/American Heart Association; BP, blood pressure; DBP, diastolic blood pressure; ESC/ESH, European Society of Cardiology/European Society of Hypertension; ESH, European Society of Hypertension; HBPM, home blood pressure monitoring; IDH, isolated diastolic hypertension; ISH (guideline), International Society of Hypertension; SBP, systolic blood pressure.

**Figure 1:**
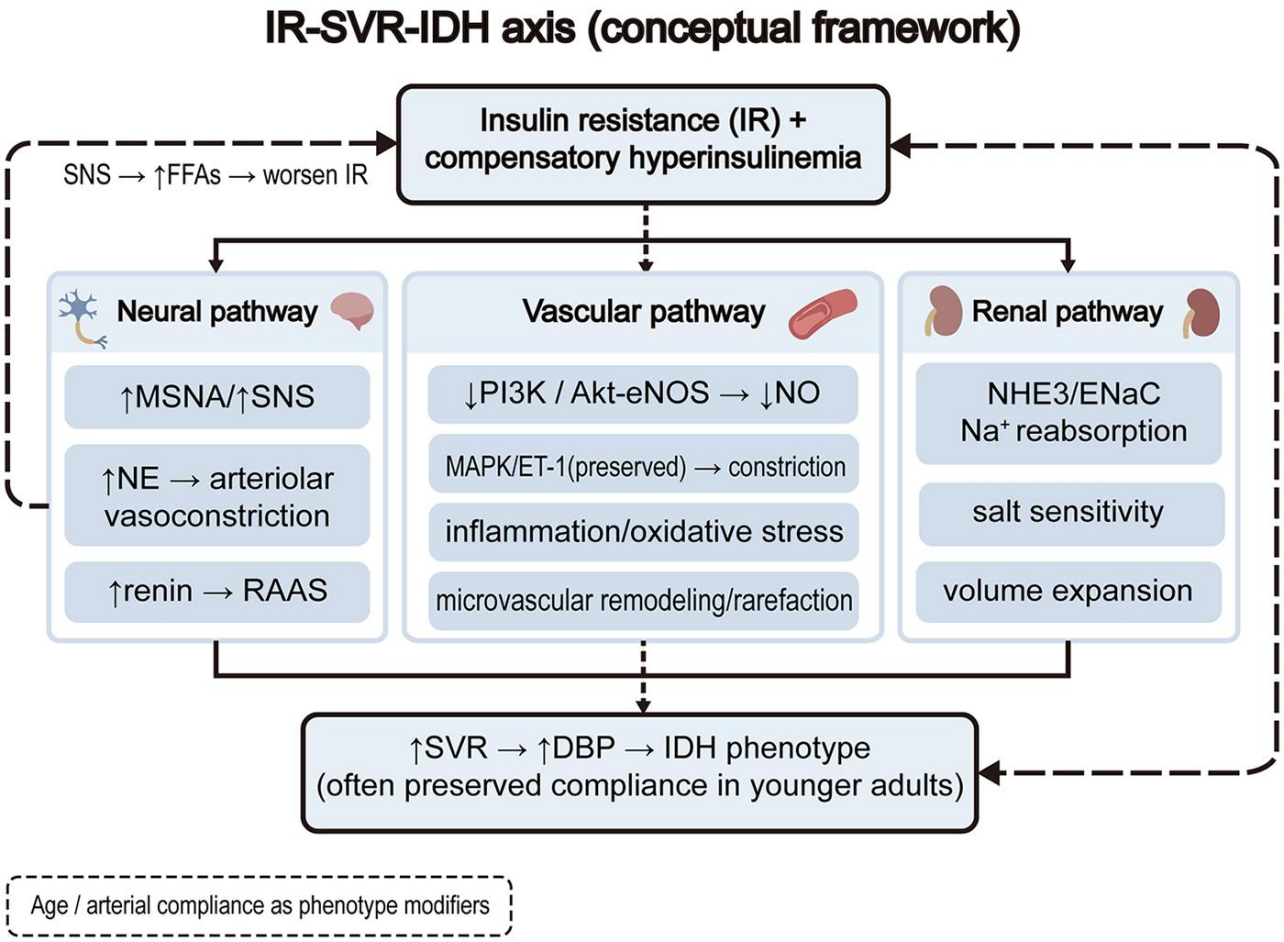
The IR–SVR–IDH axis (conceptual framework). Insulin resistance (IR) with compensatory hyperinsulinemia may bias the circulation toward higher systemic vascular resistance (SVR) via three interacting pathways: (i) neural—sympathetic activation with downstream renin–angiotensin–aldosterone system (RAAS) engagement; (ii) vascular—impaired endothelial nitric oxide (NO)-mediated vasodilation, reinforced by inflammation/oxidative stress and microvascular remodeling/rarefaction; and (iii) renal—relatively preserved sodium-retaining actions (e.g., NHE3/ENaC), promoting salt sensitivity and volume expansion. These pathways converge on increased diastolic blood pressure (DBP) and the isolated diastolic hypertension (IDH) phenotype, particularly in younger adults with relatively preserved aortic compliance. Dashed arrows indicate reinforcing feedback loops. IR, insulin resistance; SVR, systemic vascular resistance; IDH, isolated diastolic hypertension; RAAS, renin–angiotensin–aldosterone system; NO, nitric oxide; NHE3, Na+/H+ exchanger 3; ENaC, epithelial sodium channel; DBP, diastolic blood pressure.

Despite being relatively common, the prognostic significance of IDH and its optimal management remain controversial, and guideline recommendations are limited or inconsistent (6–8). This controversy is partly driven by a fundamental question: which threshold defines IDH in a way that is clinically meaningful and consistent across populations? Lowering the diagnostic threshold (e.g., the ACC/AHA definition with DBP ≥ 80 mmHg) increases the frequency with which IDH is identified, whereas the ESC/ESH approach preserves the traditional DBP ≥ 90 mmHg cutoff; consequently, studies and clinical decisions are only partially comparable across definitions (1, 2) (Table 1). To facilitate integration of mechanistic and clinical evidence across these definitional boundaries, we summarize the proposed IR–SVR–IDH axis as a unifying framework in Figure 1.

Importantly, multiple studies report significant associations between IDH and adverse cardiovascular events in young and middle-aged populations, supporting the need to take this phenotype seriously (9–12). However, the evidence is not fully consistent. Studies in the United States applying the 2017 ACC/AHA definition of IDH did not observe a significant association with atherosclerotic outcomes, whereas large cohorts such as the UK Biobank reported a higher risk of composite cardiovascular events, potentially more pronounced in younger individuals and women (13, 14). In an ultra-large cohort of young Asian adults, stage 1 IDH defined by the 2017 ACC/AHA criteria was associated with increased cardiovascular event risk (15). Furthermore, systematic reviews and meta-analyses based on the 2018 ESC/ESH criteria suggest that the risk association of IDH is more consistent in younger and Asian populations (16). Taken together, IDH appears to be a heterogeneous blood pressure phenotype: its risk magnitude may depend on diagnostic thresholds, age and region, the underlying cardiometabolic context, and whether it represents persistent IDH rather than isolated elevated readings (Table 1).

**Figure 2:**
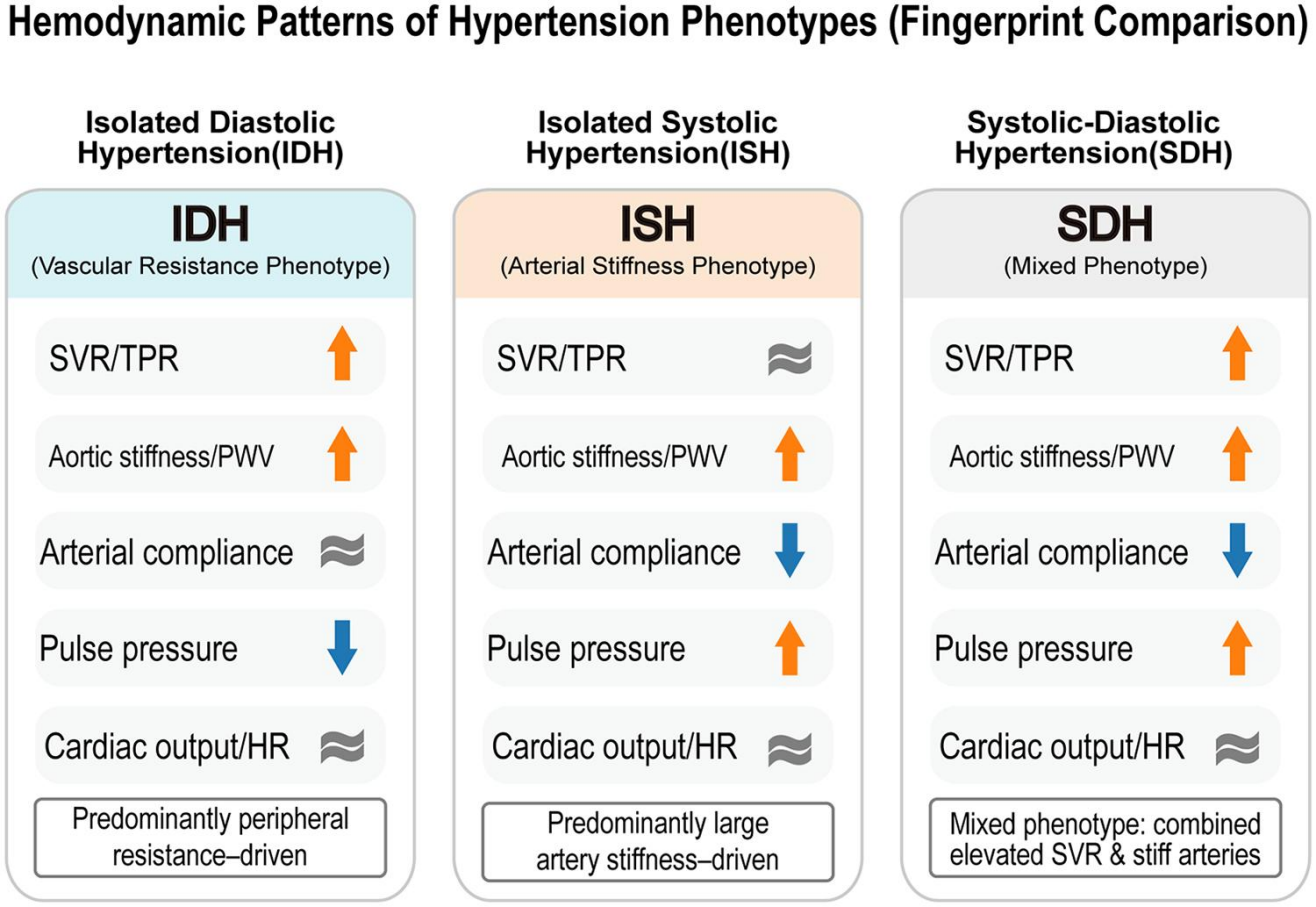
Hemodynamic fingerprints across hypertension phenotypes. Schematic comparison of isolated diastolic hypertension (IDH), isolated systolic hypertension (ISH), and systolic–diastolic hypertension (SDH). IDH is typically characterized by higher systemic/total peripheral resistance (SVR/TPR) with relatively preserved large-artery compliance and a narrower pulse pressure (PP); cardiac output/heart rate (CO/HR) is near normal or mildly elevated (heterogeneous subset). ISH is predominantly driven by increased aortic stiffness (higher pulse wave velocity, PWV) and reduced arterial compliance, yielding widened PP. SDH commonly reflects combined increases in resistance and large-artery stiffness. Arrows indicate direction of change relative to normotension (↑ increased; ↓ decreased; ≈ near-normal), emphasizing phenotype heterogeneity rather than absolute values. IDH, isolated diastolic hypertension; ISH, isolated systolic hypertension; SDH, systolic–diastolic hypertension; SVR/TPR, systemic/total peripheral resistance; PWV, pulse wave velocity; PP, pulse pressure; CO, cardiac output; HR, heart rate.

From a pathophysiological perspective, the hemodynamic profile of IDH is particularly informative. Unlike isolated systolic hypertension (ISH), which is largely attributable to age-related arterial stiffening, IDH is often characterized by increased systemic vascular resistance (SVR) with relatively preserved aortic compliance (17, 18) (Figure 2, Table 2). This pattern suggests that core mechanisms in many IDH presentations may primarily operate at the level of resistance vessels (19, 20). Yet, in real-world practice, IDH remains underdiagnosed and undertreated, potentially missing opportunities for early intervention (21, 22). A related practical issue is that IDH frequently occurs within relatively modest DBP elevations, where single or infrequent office measurements are more vulnerable to measurement error, the white-coat effect, or masked hypertension. Accordingly, multiple guidelines and practice documents emphasize confirmation of the diagnosis and improvement of phenotypic accuracy using ambulatory blood pressure monitoring (ABPM) or home blood pressure monitoring (HBPM) whenever feasible (1, 23, 24) (Table 1).

**Table 2:** Key studies describing hemodynamic patterns in IDH compared with other hypertension phenotypes.

Study (year)	Population/design	Phenotype definition (symbolic)	Hemodynamic assessment	Main signal for IDH (vs. comparators)	Pitfalls/boundaries
Aristizábal-Ocampo et al. (2023) (35)	Adults; untreated; cross-sectional	ABPM-IDH: SBP < 130 & DBP ≥ 80; PP < 50; comparators: NT/ISH/SDH (ABPM)	ABPM + model-derived (Windkessel) estimates	Narrow PP; compliance ∼preserved; resistance-leaning (TPR↑)	Model-based; PP cut selects narrow PP subtype
Álvarez-Montoya et al. (P165 abstract, 2024) (38)	ABPM clinic sample; untreated; cross-sectional	ABPM-IDH: 24 h DBP > 80 (SBP criterion per abstract)	ABPM + estimated hemodynamics; clustering	Two profiles: cardiogenic (↑HR/↑CI) vs. vasotonic (↑SVR)	Abstract-only; referral bias; methods limited
Romero et al. (2013) (37)	Clinic-referred non-medicated hypertensive patients; re-analysis; ABPM-screened young PDH/IDH subset; observational	Age <50 years; daytime pulse pressure ≤45 mmHg (ABPM); PDH/IDH spectrum (narrow PP concept)	Impedance cardiography	Marked heterogeneity: hyperdynamic vs. vasotonic (CI/SVR combinations)	Selected phenotype; lab protocols differ
Romero et al. (2021) (27)	Critical/narrative review	Conceptual: PDH (narrow PP) vs. PSH (wide PP)	Evidence synthesis	Frames IDH/PDH as resistance-vessel–dominant vs. ISH stiffness-dominant	Not primary data; conceptual framing
Saarinen et al. (2025) (28)	Adults; observational	PDH vs. PSH: aortic BP-based classification	Tonometry + impedance cardiography (incl. tilt)	PDH shows more hyperdynamic features (↑HR/↑CO); SVR patterns vary	Not strict IDH definition; specialized methods

(1) Table summarizes directional hemodynamic signals and heterogeneity (not pooled quantitative estimates). (2) Definitions vary (office vs. ABPM; PP cuts), which can materially shift who is classified as IDH. (3) Evidence levels differ across entries; in particular, conference abstracts/supplement items should be interpreted as preliminary and hypothesis-generating. ABPM, ambulatory blood pressure monitoring; CI, cardiac index; CO, cardiac output; DBP, diastolic blood pressure; IDH, isolated diastolic hypertension; ISH, isolated systolic hypertension; NT, normotension; PDH, predominantly diastolic hypertension; PP, pulse pressure; PSH, predominantly systolic hypertension; SDH, systolic–diastolic hypertension; SVR/TPR, systemic/total peripheral resistance.

During our literature review, we noted growing evidence linking insulin resistance (IR) to IDH. Traditionally viewed as a core metabolic abnormality in type 2 diabetes and metabolic syndrome (MetS), IR has vascular and neurohumoral consequences that extend well beyond glucose regulation (25). At the vascular level, IR is associated with impaired endothelial nitric oxide signaling, chronic low-grade inflammation, sympathetic nervous system overactivation, and abnormal renal sodium handling—each of which may plausibly contribute to increased peripheral resistance (26). When these mechanisms cluster, as often occurs in metabolically unhealthy individuals, their combined effect may sustain SVR elevation and manifest clinically as higher DBP. From this perspective, IDH may reflect not merely an isolated blood pressure abnormality but a vascular manifestation of underlying metabolic dysfunction (Figure 1).

**Figure 3:**
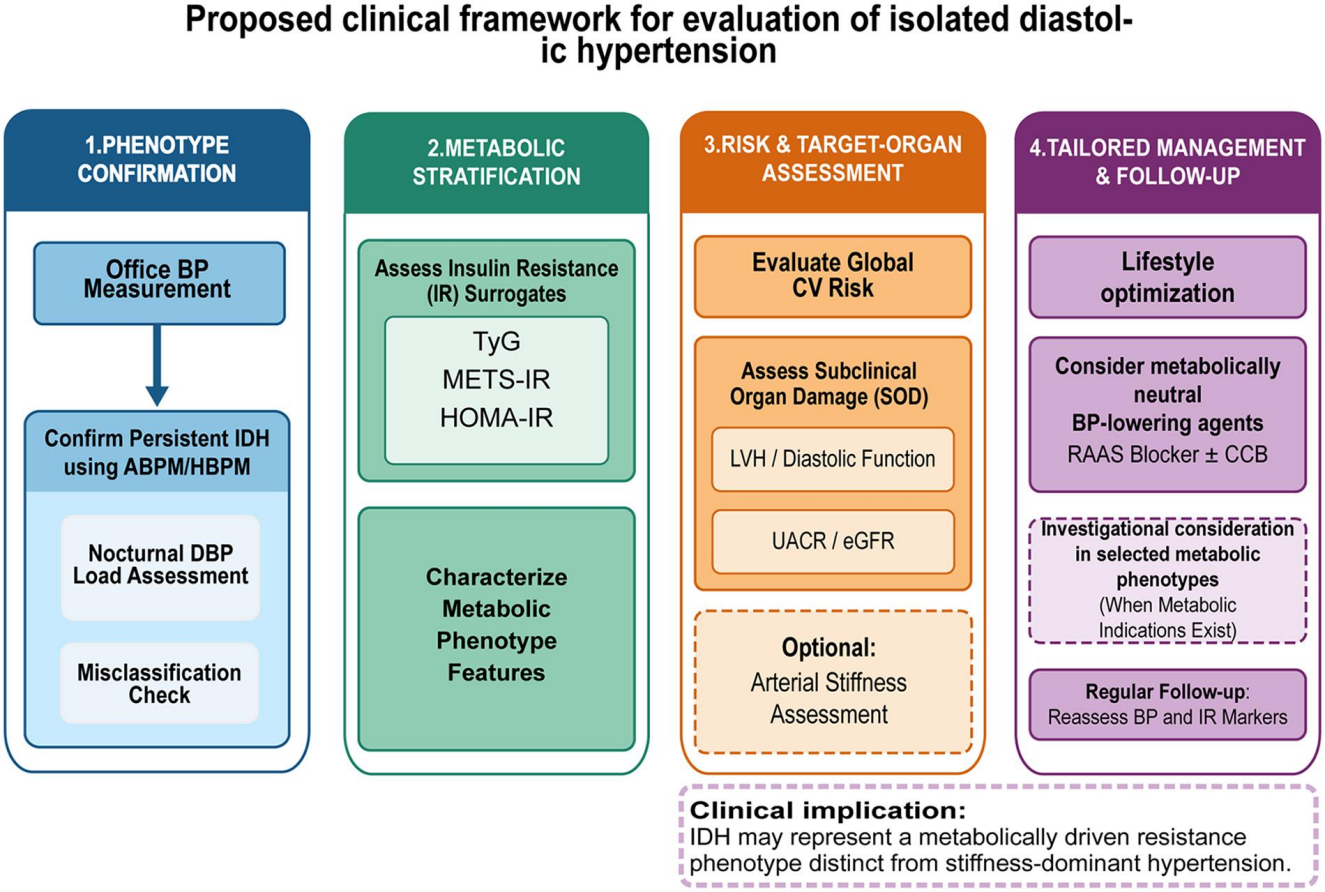
Workflow to identify and manage a metabolically driven IDH phenotype. Stepwise approach to (i) confirm persistent IDH using out-of-office blood pressure (ABPM/HBPM), including nocturnal DBP load assessment and misclassification check; (ii) stratify metabolic drive using insulin resistance (IR) surrogates (e.g., TyG, METS-IR, HOMA-IR) alongside clinical metabolic phenotype features; (iii) assess overall cardiovascular risk and early subclinical target-organ damage (e.g., LVH/diastolic dysfunction, albuminuria/renal function), with arterial stiffness assessment as an optional component; and (iv) implement tailored management prioritizing lifestyle optimization and metabolically neutral antihypertensive regimens (e.g., RAAS blockade ± calcium channel blockers), with cardiometabolic therapies considered when metabolic indications exist, plus regular follow-up to reassess BP and IR markers. Solid boxes denote core steps; dashed boxes indicate optional/indication-dependent components. Clinical implication: IDH may represent a metabolically driven resistance phenotype distinct from stiffness-dominant hypertension. ABPM, ambulatory blood pressure monitoring; HBPM, home blood pressure monitoring; IR, insulin resistance; TyG, triglyceride–glucose index; METS-IR, metabolic score for insulin resistance; HOMA-IR, homeostasis model assessment of insulin resistance; RAAS, renin–angiotensin–aldosterone system; LVH, left ventricular hypertrophy; UACR, urine albumin-to-creatinine ratio; eGFR, estimated glomerular filtration rate; CCB, calcium channel blocker; DBP, diastolic blood pressure.

**Figure 4:**
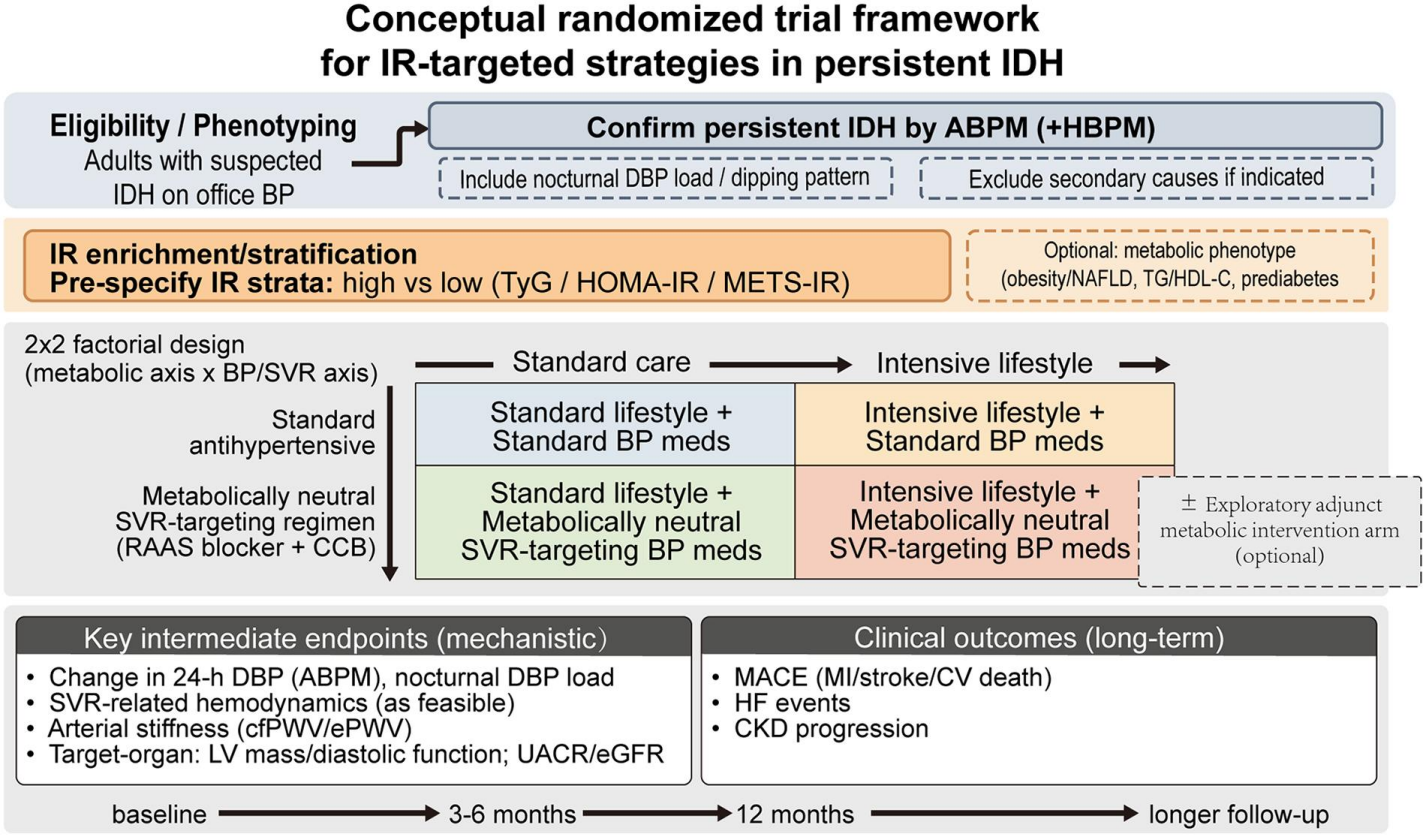
Trial blueprint for IR-targeted strategies in ABPM-confirmed persistent IDH. Conceptual design for future randomized trials that enroll adults with office-suspected IDH, confirm persistent phenotype via ABPM (±HBPM) including nocturnal DBP load assessment, and exclude secondary causes when indicated. Participants are enriched and stratified by insulin resistance (IR) burden using pragmatic surrogates (e.g., TyG, HOMA-IR, METS-IR), with optional metabolic phenotyping (e.g., obesity/NAFLD, TG/HDL-C, prediabetes). A 2 × 2 factorial design tests integrated interventions targeting both metabolic and resistance-vessel pathways: combining standard vs. intensive lifestyle modification with standard vs. metabolically neutral, SVR-oriented antihypertensive regimens (e.g., RAAS blockade ± calcium channel blockers), plus optional cardio-metabolic therapy when indicated. Suggested endpoints include mechanistic intermediate outcomes (changes in 24-h/nocturnal DBP, SVR-related hemodynamics, arterial stiffness, and target-organ damage) assessed at 3–6 months, and adjudicated long-term clinical outcomes (e.g., MACE, heart failure events, CKD progression) with follow-up through 12 months and beyond. Solid boxes represent core components; dashed boxes indicate optional elements. ABPM, ambulatory blood pressure monitoring; HBPM, home blood pressure monitoring; IDH, isolated diastolic hypertension; IR, insulin resistance; TyG, triglyceride–glucose index; METS-IR, metabolic score for insulin resistance; HOMA-IR, homeostasis model assessment of insulin resistance; SVR, systemic vascular resistance; RAAS, renin–angiotensin–aldosterone system; CCB, calcium channel blocker; DBP, diastolic blood pressure; MACE, major adverse cardiovascular events; CKD, chronic kidney disease; NAFLD, non-alcoholic fatty liver disease; TG/HDL-C, triglyceride to high-density lipoprotein cholesterol ratio; LV, left ventricular; UACR, urine albumin-to-creatinine ratio; eGFR, estimated glomerular filtration rate; cfPWV/ePWV, carotid-femoral/estimated pulse wave velocity.

Against this background, this review synthesizes current evidence linking IR to IDH. We propose the IR–SVR–IDH axis as an integrative framework that organizes how IR may promote diastolic hypertension through interacting neural, vascular, and renal pathways (Figure 1). We first summarize the hemodynamic fingerprints that distinguish IDH from other hypertension phenotypes (Figure 2, Table 2), then evaluate clinically practical IR surrogates and representative population evidence linking IR burden to IDH risk (Tables 3, 4). Finally, we discuss management implications using a stepwise workflow for identifying a metabolically driven IDH phenotype (Figure 3) and outline trial design priorities to test whether IR-targeted strategies improve hemodynamics and long-term outcomes in ABPM-confirmed persistent IDH (Figure 4), particularly in younger individuals who may benefit most from early identification and intervention.

**Table 3:** Practical insulin resistance (IR) surrogates for identifying a metabolically driven IDH phenotype.

Index	Simplified mathematical expression	Inputs	Best use (IDH context)	Strengths	Pitfalls/boundaries
HOMA-IR	FPG×FI/22.5	FI, FPG	Stratify or confirm IR (when insulin available)	Classic; intuitive	Insulin assay variability; fasting-only
TyG	ln(TG×FPG/2)	TG, FPG	Screen and stratify metabolic IDH	Cheap; scalable	Unit-dependent; TG fluctuates and illness sensitive
METS-IR	ln(2×FPG+TG)×BMI/ln(HDL-C)	FPG, TG, HDL-C, BMI	Enrich and stratify in cohorts or trials	Adds adiposity and lipids	Unit-dependent; BMI and HDL confounding
TG/HDL-C	TG/HDL−C	TG, HDL-C	Quick adjunct marker	Very simple	Sex and ethnicity effects; diet and alcohol sensitive
TyG-BMI	TyG×BMI	TyG, BMI	Refine stratification (obesity prominent)	Often improves discrimination	Inherits TyG limits; BMI ≠ visceral fat
TyG-WC	TyG×WC	TyG, WC	Refine stratification (central obesity)	Adds central adiposity	WC measurement error; ethnicity differences
Matsuda index	$10,000/\mathrm{sqrt}(FPG\times FI\times G_{\mathrm{mean}}\times I_{\mathrm{mean}})$	OGTT glucose and insulin	Research phenotyping	Dynamic, physiology-rich	Not routine; protocol-dependent

(1) Simplified mathematical expressions are shown for readability. Full formulas are presented as originally defined in the corresponding source publications (31, 32, 150, 151). (2) For TyG, METS-IR, and Matsuda index, glucose and lipid concentrations are expressed in mg/dL, consistent with their original definitions. (3) For HOMA-IR, fasting plasma glucose (FPG) is expressed in mmol/L and fasting insulin (FI) in µU/mL. (4) ln denotes natural logarithm; sqrt denotes square root. (5) These indices are intended for metabolic risk stratification or mechanistic enrichment and should not be interpreted as diagnostic criteria for isolated diastolic hypertension (IDH). Full formulas (as originally defined): HOMA-IR = [FPG (mmol/L) × FI (µU/mL)]/22.5; TyG index = ln [TG (mg/dL) × FPG (mg/dL)/2]; METS-IR = {ln [(2 × FPG (mg/dL) + TG (mg/dL))] × BMI (kg/m^2^)}/ln [HDL-C (mg/dL)]; TG/HDL-C ratio = TG/HDL-C (same unit required); TyG-BMI = TyG × BMI (kg/m^2^); TyG-WC = TyG × WC (cm); Matsuda index = 10,000/sqrt{[FPG (mg/dL) × FI (µU/mL)] × [mean glucose (mg/dL) × mean insulin (µU/mL)]}. BMI, body mass index; FI, fasting insulin; FPG, fasting plasma glucose; HDL-C, high-density lipoprotein cholesterol; HOMA-IR, homeostasis model assessment of insulin resistance; IDH, isolated diastolic hypertension; IR, insulin resistance; METS-IR, metabolic score for insulin resistance; OGTT, oral glucose tolerance test; TG, triglycerides; TyG, triglyceride–glucose index; WC, waist circumference.

**Table 4:** Representative clinical studies linking IR surrogates to IDH prevalence/incidence and risk stratification.

Study (year)	Region	Population/design	IDH definition	IR metric(s)	Key finding (adjusted)	Evidence tier	Pitfalls/boundaries
Cai et al. (2022) (88)	China	Adults (*n* = 16,793); cross-sectional	SBP < 140 & DBP ≥ 90 (office)	TyG	TyG (continuous): IDH OR 2.94 (1.66–5.23); stronger in women (female OR 6.58 vs. male 1.77)	C	Cross-sectional; single province; office BP
CHIEF cohort (Tsai et al., 2024) (86)	Taiwan	Military 18–39; prospective (2014–2020)	Stage 1 IDH: SBP < 130 & DBP 80–89 (office)	TyG; TG/HDL-C; METS-IR	Incident stage 1 IDH (per 1-unit): TyG HR 1.376 (1.123–1.687); TG/HDL-C HR 1.082 (1.039–1.127); METS-IR HR 3.455 (1.921–6.214)	B	Mostly young/fit; no ABPM; per-unit scales differ
CNHHS 2017 (Front Endocrinol; 2025)	China	Children/adolescents 7–17 (*n* = 12,087); cross-sectional	Pediatric IDH: DBP ≥ 95th & SBP < 95th percentile	TyG; METS-IR	Per 1-unit: TyG IDH OR 1.47 (1.21–1.79); METS-IR IDH OR 1.06 (1.03–1.08)	C	Pediatric definition; subgroup heterogeneity
Sakoda et al. (2024; Hypertens Res) (95)	Japan	Middle-aged men (*n* = 41,811); cohort	140/90 threshold groups (includes IDH)	TyG	Within IDH group: per-unit TyG predicts incident CKD HR 1.31 (1.06–1.62)	B	Men only; health-check population; kidney endpoint
Jones et al. (2026; NHANES) (94)	USA	NHANES adults; cohort with mortality follow-up	SBP < 130 & DBP ≥ 80 (office)	DM status	DM−: NS; DM+: ↑CVD/AC mortality; interaction *P* < 0.01	B	Office BP; observational

(1) Effect estimates are reported as adjusted ORs/HRs (95% CI) using the original study scaling (e.g., continuous/per 1-unit increase or subgroup comparisons). (2) IDH definitions vary across studies by threshold and BP ascertainment (predominantly office BP in the studies listed), contributing to heterogeneity. (3) NHANES HR details (Jones et al.): DM− IDH: CVD mortality HR 0.76 (*P* = 0.32), all-cause HR 0.86 (*P* = 0.20); DM+ IDH: CVD mortality HR 3.65 (*P* < 0.01), all-cause HR 1.97 (*P* < 0.01); subtype × DM interaction *P* < 0.01. DM−/DM+ denote participants without/with diabetes; NS denotes not significant; AC denotes all-cause. (4) Evidence tiers: Tier A, human physiological/hemodynamic studies; Tier B, prospective cohorts; Tier C, cross-sectional/retrospective studies; Tier D, preclinical/*in vitro*. ABPM, ambulatory blood pressure monitoring; CKD, chronic kidney disease; CNHHS, China National Nutrition and Health Surveillance; CVD, cardiovascular disease; DBP, diastolic blood pressure; DM, diabetes mellitus; HR, hazard ratio; IDH, isolated diastolic hypertension; IR, insulin resistance; METS-IR, metabolic score for insulin resistance; NHANES, National Health and Nutrition Examination Survey; NS, not significant; OR, odds ratio; SBP, systolic blood pressure; TG, triglycerides; TyG, triglyceride–glucose index.

Heterogeneity of IDH and rationale for an IR-focused framework. Importantly, IDH is a heterogeneous blood pressure phenotype rather than a single disease entity. Beyond IR-enriched presentations, elevated DBP with relatively preserved SBP may arise from partially overlapping drivers such as increased resistance-vessel tone with hyperdynamic features, salt sensitivity with variable metabolic burden, and secondary contributors that merit clinical consideration (e.g., renovascular or other secondary hypertension etiologies) (27–30). Moreover, because borderline DBP elevations are susceptible to misclassification in office settings, careful phenotype confirmation with ABPM/HBPM remains essential before etiologic inference or treatment escalation (24). In this context, we focus on an IR-enriched, high-SVR pathway because IR-related abnormalities are common in young and middle-aged populations, mechanistically converge on SVR elevation, and can be pragmatically identified using scalable surrogate indices (e.g., TyG and METS-IR) that support actionable cardiometabolic risk stratification (31, 32) (Figure 3, Table 3). This IR–SVR–IDH framework is intended to complement—rather than replace—broader etiologic evaluation and rigorous BP phenotyping (Table 1).

### 1.1 Search strategy and scope of this narrative review

We conducted a focused literature search to inform this narrative review on isolated diastolic hypertension (IDH), insulin resistance (IR), systemic vascular resistance (SVR), and related mechanistic and clinical evidence. We searched PubMed/MEDLINE from database inception to May 9, 2026, using combinations of terms including isolated diastolic hypertension, diastolic blood pressure, systemic vascular resistance/total peripheral resistance, insulin resistance, hyperinsulinemia, triglyceride–glucose (TyG) index, METS-IR, HOMA-IR, sympathetic nervous system, endothelial dysfunction, renin–angiotensin–aldosterone system, salt sensitivity, and ambulatory blood pressure monitoring. We also screened reference lists of key reviews and primary studies to identify additional relevant publications.

Given heterogeneity in IDH definitions and the predominance of observational and mechanistic evidence in this field, we selected studies to represent the breadth of evidence supporting a pathway-based synthesis. We prioritized human studies with clear BP phenotyping (including ABPM/HBPM when available), prospective designs for outcome associations, and physiological/hemodynamic studies relevant to SVR, while using animal and *in vitro* evidence to support biological plausibility when appropriate. This article is a narrative (non-systematic) review; we did not perform a formal risk-of-bias scoring for all included studies, nor did we conduct a meta-analysis.

### 1.2 Evidence grading and interpretation in this narrative review

The evidence base relevant to IDH, IR, and SVR is heterogeneous in study design, BP phenotyping (office vs. ABPM/HBPM), and endpoints. To improve interpretability within a narrative (non-systematic) synthesis, we graded evidence using a pragmatic tiering approach and aligned the strength of language with evidence strength. Tier A comprised human physiological/hemodynamic studies and mechanistic clinical investigations directly informing SVR regulation (e.g., autonomic measures, vascular function, salt-sensitivity phenotyping). Tier B comprised prospective cohort studies evaluating incident IDH or longitudinal clinical outcomes. Tier C comprised cross-sectional analyses and retrospective observational studies, which were used primarily to support association patterns and hypothesis generation rather than causal inference. Tier D comprised preclinical or *in vitro* evidence supporting biological plausibility. Across sections, we preferentially emphasized ABPM/HBPM-confirmed phenotypes when available and highlighted key limitations (e.g., residual confounding, single-visit office BP misclassification, and definition heterogeneity) when interpreting observational findings.

## 2 Hemodynamic characteristics of isolated diastolic hypertension

Before discussing how insulin resistance contributes to the IR–SVR–IDH axis, it is essential to clarify the hemodynamic fingerprint that distinguishes IDH from other hypertension subtypes (Figure 2, Table 2). Isolated systolic hypertension (ISH) predominantly affects older adults and is driven mainly by age-related stiffening of large elastic arteries and increased pulse wave velocity (33, 34). In contrast, IDH more often presents with relatively preserved large-artery (aortic) compliance—particularly in younger individuals—despite persistently elevated DBP. Clinically, this is frequently accompanied by a relatively narrow pulse pressure, supporting the view that the dominant abnormality lies in increased resistance-vessel (small-artery/arteriolar) tone rather than isolated large-artery stiffening. Here, SVR is used as the primary term [also referred to as total peripheral resistance (TPR) in many hemodynamic models], whereas peripheral vascular resistance (PVR) is often reported in non-invasive or model-based assessments and broadly tracks resistance-vessel load.

This distinction has implications for both mechanism and natural history. A review proposed that a predominantly diastolic pattern accompanied by a narrow pulse pressure may represent a more resistance-mediated subtype, potentially evolving over time into typical systolic–diastolic hypertension (27). Building on this concept, defining diastolic-dominant individuals by narrow pulse pressure (e.g., ≤45 mmHg) places IDH and some narrow–pulse-pressure systolic–diastolic hypertension on a continuum, which may facilitate interpretation of progression and improve risk stratification (27). Accordingly, rather than treating IDH as a completely static and independent category, it may be more appropriate to view it as a stage/subphenotype in which resistance-vessel abnormalities predominate.

Hemodynamic stratification using ABPM provides further support. In IDH, total arterial compliance often approximates that of normotensive controls, whereas hemodynamic alterations are more evident at the resistance-vessel level (e.g., higher peripheral resistance/small-artery tone), with mildly increased cardiac output or cardiac index in a subset of individuals (35) (Table 2). In addition, unsupervised clustering of conventional ABPM features can identify a circulatory phenotype characterized primarily by elevated DBP and higher vascular resistance, indicating that ambulatory monitoring can capture mechanism-relevant information beyond blood pressure levels alone (36).

Findings from the ACCT (Anglo-Cardiff Collaborative Trial) cohort further reinforce this phenotype-based distinction. Across standardized measurements, IDH has been linked more strongly to elevated PVR, whereas systolic–diastolic hypertension more often shows combined increases in PVR and pulse wave velocity (PWV), with patterns that may vary by age and sex (17) (Table 2). Consistently, studies focusing on predominantly diastolic hypertension suggest that diastolic-dominant individuals may exhibit higher heart rate and cardiac index than predominantly systolic individuals; although both groups can show elevated SVR, the magnitude and combination of hemodynamic abnormalities can differ substantially (28). Collectively, these data suggest that IDH commonly carries a greater resistance-vessel burden, but it should not be regarded as a homogeneous entity.

The value of this hemodynamic framework for the subsequent sections is that it helps prioritize plausible upstream drivers of IDH—factors that can chronically increase small-artery tone and peripheral resistance, often overlapping with sympathetic activation, volume load, or mildly hyperdynamic circulation. As discussed later, insulin resistance converges on the shared endpoint of elevated SVR through interacting neural, vascular, and renal pathways, thereby providing a mechanistic basis for the IR–SVR–IDH axis that aligns closely with the observed hemodynamic phenotype (Figure 1). Key hemodynamic evidence supporting these phenotype patterns is summarized in Table 2 (17, 27, 28, 35–38).

## 3 Mechanistic link between insulin resistance and elevated SVR: a pathway-based view of the IR–SVR–IDH axis

This axis can be summarized as a metabolically driven cascade in which insulin resistance (IR), together with compensatory hyperinsulinemia, biases the circulation toward a high-resistance state through neural, vascular, and renal mechanisms, thereby predisposing to a phenotype dominated by elevated diastolic pressure (39) (Figure 1). The strongest support for this concept comes from human physiology and mechanistic studies implicating pathways such as IR/hyperinsulinemia → sympathetic activation, endothelial dysfunction, and enhanced renal sodium reabsorption. However, the extent to which these alterations determine IDH onset and long-term outcomes is still inferred largely from observational evidence, and population-level modifiers (e.g., age, adiposity/inflammatory burden, salt intake, and baseline sympathetic tone) likely shift the relative weights of these pathways (40). Therefore, the IR–SVR–IDH axis is best viewed as a probabilistic, context-dependent framework rather than a one-size-fits-all mechanism.

### 3.1 Neural mechanism: hyperinsulinemia-driven sympathetic overactivation

Sympathetic nervous system (SNS) activity is a major determinant of resistance-vessel tone and SVR, making it a plausible upstream driver of diastolic pressure elevation. Insulin is not only a metabolic hormone; it also exerts central sympathoexcitatory actions. Under physiological conditions, insulin crosses the blood–brain barrier and acts on hypothalamic circuits to increase muscle sympathetic nerve activity (MSNA) (41). In insulin-sensitive individuals, insulin also exerts direct peripheral vasodilatory actions, and the net hemodynamic response to acute hyperinsulinemia often results in minimal changes in arterial pressure due to counterbalancing vascular and autonomic/cardiac responses (42–44). In insulin-resistant states, this balance may be disrupted in a direction that favors vasoconstriction. The concept of selective insulin resistance has gained prominence, in which peripheral tissues (e.g., skeletal muscle) exhibit impaired metabolic responses to insulin while central pathways may retain sensitivity to insulin's sympathoexcitatory effects (45–47). As a consequence, compensatory hyperinsulinemia may chronically activate the SNS without an adequate vasodilatory counter response.

This neurogenic shift has several downstream effects that are directly relevant to IDH. Increased norepinephrine release activates α1-adrenergic receptors on resistance arterioles, promoting sustained vasoconstriction and higher SVR (48, 49). Sympathetic activation can also stimulate renal renin release and stimulate the renin–angiotensin–aldosterone system (RAAS), further promoting vasoconstriction and sodium retention. In parallel, SNS-driven lipolysis increases circulating free fatty acids (FFAs), which can worsen IR, reinforcing a feed-forward loop between metabolic dysfunction and neurogenic vasoconstriction (49, 50). Evidence from human physiological studies—particularly those using MSNA and reflex regulation endpoints—supports the direction that sympathetic excitation is more likely in a high-insulin/IR context (51). However, the net effect on DBP/SVR is strongly modified by endothelial vasodilatory reserve, salt intake, and baseline sympathetic tone, making this pathway more relevant for metabolically burdened individuals with heightened sympathetic responsiveness rather than all IDH presentations (40).

### 3.2 Vascular mechanism: endothelial insulin resistance and the inflammation–oxidative stress axis

Endothelial insulin resistance provides a direct vascular route by which IR can reduce resistance-vessel vasodilatory reserve and increase SVR. Under normal conditions, insulin signaling in endothelial cells activates the insulin receptor substrate (IRS)–PI3K/Akt pathway, leading to phosphorylation of endothelial nitric oxide synthase (eNOS) and nitric oxide (NO) production (25, 48). NO then diffuses to vascular smooth muscle cells (VSMCs) to induce relaxation and lower SVR. In IR, this vasodilatory arm is preferentially impaired while vasoconstrictor signaling may be relatively preserved. Inflammatory cytokines and elevated FFAs can weaken downstream PI3K/Akt signaling (52, 53), whereas the mitogen-activated protein kinase/extracellular signal-regulated kinase (MAPK/ERK) pathway supporting vasoconstrictor responses—including endothelin-1 (ET-1) production—often remains intact or is upregulated (48). The resulting signaling imbalance shifts vascular behavior away from NO-mediated relaxation toward vasoconstrictor bias, increasing SVR. Beyond acute tone, IR-related dyslipidemia, oxidative stress, and chronic low-grade inflammation can further reduce NO bioavailability and favor microvascular remodeling/rarefaction, contributing to a more fixed high-resistance state (54–57).

Mechanistic studies and review syntheses broadly support the concept that endothelial insulin resistance and impaired NO signaling reduce vasodilatory reserve at the resistance-vessel level, and they also suggest that endothelial cell mineralocorticoid receptor (ECMR)/epithelial sodium channel (ENaC)-related mechanisms may further increase microvascular resistance in MetS contexts (58, 59). However, a high-SVR background does not necessarily translate into an IDH phenotype, because its blood pressure expression is also shaped by large-artery compliance, volume status, and neurohumoral activation; thus, this pathway is best positioned as a core explanation for IR-related high SVR rather than a mechanism uniquely specific to IDH (40).

### 3.3 Renal mechanism: sodium retention, salt sensitivity, and volume expansion

Renal sodium handling represents a third pathway through which IR can sustain a high-SVR, DBP-elevating milieu. A notable feature is that even in systemic IR, insulin's sodium-retaining (antinatriuretic) actions in the kidney may be relatively preserved—another manifestation of selective insulin resistance. Insulin can enhance sodium reabsorption along multiple nephron segments, including activation of the Na+/H+ exchanger 3 (NHE3) in the proximal tubule (60) and upregulation of the epithelial sodium channel (ENaC) in the distal nephron (61). In IR, heightened SNS and RAAS activity can synergize with these direct tubular actions to promote sodium retention. The resulting volume expansion can increase preload and stroke volume and, if heart rate is maintained, can maintain or increase cardiac output, thereby contributing to higher DBP (21, 29); it can also contribute to salt sensitivity, a common feature in IR and MetS (62–64). Mechanistically, salt sensitivity in this setting may involve impaired renal NO-dependent vasodilation, oxidative stress-related reductions in NO bioavailability, and immune activation (65–68), collectively favoring sodium and water retention and sustaining DBP elevation when SVR is already high (30, 69).

Recent follow-up studies and human salt-sensitivity phenotype research suggest a potentially bidirectional relationship between salt sensitivity and IR and raise the possibility of an immune–oxidative stress signaling–IR fluctuation link (70, 71). The evidence supporting renal sodium-retaining effects of insulin and salt-sensitive physiology is mechanistically plausible and increasingly supported by human phenotype studies linking salt sensitivity, immune/oxidative signals, and IR-related traits (29, 30, 60–71). Nevertheless, direct quantitative evidence specific to IDH remains limited, so renal/salt-sensitive mechanisms are best framed as modifiers/amplifiers—especially in metabolically burdened or salt-sensitive subgroups—rather than obligatory pathways for all IDH (40).

### 3.4 Integrating pathways: why SVR elevation can present as IDH in the young

An integrative view helps reconcile why IR can yield a DBP-dominant phenotype in some individuals but not others. Broadly, the neural pathway tends to increase resistance-vessel tone, the vascular pathway tends to reduce vasodilatory reserve and promote remodeling, and the renal pathway tends to raise the volume and salt-sensitive baseline. When these mechanisms co-occur in the same metabolic context, they more readily produce sustained SVR elevation. In younger individuals with relatively preserved large-artery compliance, this hemodynamic shift is more likely to manifest primarily as DBP elevation (Figure 1). This mechanism heterogeneity motivates phenotype-aware clinical assessment using out-of-office blood pressure monitoring and pragmatic IR surrogates, which can be translated into risk stratification and tailored management (Tables 1, 3, Figure 3). From a translational standpoint, the same logic supports trials that enroll ABPM-confirmed persistent IDH and pre-specify IR-based enrichment/stratification to test whether IR-targeted multi-component strategies improve SVR-related hemodynamics and long-term outcomes (Figure 4).

## 4 Clinical phenotypes and diagnostic significance of insulin resistance-related IDH

The IR–SVR–IDH axis provides a pathophysiological rationale for how metabolic dysfunction can translate into elevated diastolic blood pressure. This section focuses on (i) the clinical and metabolic phenotype commonly accompanying IR-related IDH, (ii) the diagnostic and risk-stratification utility of IR surrogate markers, and (iii) the prognostic implications of concurrent metabolic abnormalities. Practical IR surrogates and their required inputs are summarized in Table 3, representative population evidence is mapped in Table 4, and a stepwise workflow for phenotype confirmation, metabolic stratification, and tailored management is outlined in Figure 3 (with key phenotyping considerations in Table 1).

### 4.1 Clinical phenotype and metabolic profile

In routine practice, IDH in the setting of insulin resistance rarely appears as an isolated elevation in diastolic blood pressure. More often, it coexists with features of MetS, consistent with the systemic nature of the IR–SVR–IDH axis. Patients are frequently young-to-middle-aged adults with central obesity, hypertriglyceridemia, reduced HDL cholesterol, impaired fasting glucose, and compensatory hyperinsulinemia (72). Animal models and clinical cohorts have shown that IR commonly clusters with hypertension and dyslipidemia and is linked to cardiac and vascular remodeling that may contribute to diastolic dysfunction (73–75). Interpretation of these patterns requires attention to methodological heterogeneity: differences in IDH thresholds (80 vs. 90 mmHg) and blood pressure ascertainment (office vs. ABPM/HBPM) can meaningfully influence prevalence and risk estimates, particularly when IDH occurs in borderline ranges (Table 1).

Beyond classic MetS manifestations, IR-related IDH is also reported in high-IR populations that deserve focused attention. In women with polycystic ovary syndrome (PCOS), IR correlates with abnormal ambulatory blood pressure profiles—particularly higher diastolic pressure—highlighting the coupling between reproductive–metabolic dysfunction and cardiovascular risk in this cohort (76, 77). Across cohorts, higher IR indices such as HOMA-IR correlate positively with DBP levels (77, 78), and visceral obesity has been identified as an important predictor of both IR and hypertension (79). In addition, IR-associated IDH may coincide with subclinical cardiac involvement even before overt cardiovascular disease becomes apparent; left ventricular hypertrophy and impaired diastolic relaxation have been detected despite preserved ejection fraction (75, 80). Collectively, these observations support viewing IR-associated IDH as a cardiometabolic phenotype that may already be linked to early target-organ involvement (81, 82), although most evidence is observational and sensitive to age, disease duration, and whether IDH is persistent (particularly when confirmed by ABPM).

### 4.2 Application of insulin resistance markers in IDH diagnosis and risk stratification

Because direct measurement of insulin sensitivity by the hyperinsulinemic–euglycemic clamp is impractical in routine care, surrogate markers are often used to identify metabolically driven IDH. These markers can be grouped into insulin-based indices requiring insulin assays and non–insulin-based indices derived from routine lipid and glucose measurements; formulas, inputs, and practical caveats are summarized in Table 3.

#### 4.2.1 Insulin-based markers

The homeostasis model assessment of insulin resistance (HOMA-IR) is among the most widely used insulin-based surrogates of hepatic/systemic insulin resistance (Table 3). In hypertensive populations (including those with IDH), HOMA-IR is often higher than in normotensive controls (83, 84), and oral glucose tolerance test (OGTT)-derived indices (e.g., the Matsuda index) may provide complementary information about peripheral insulin sensitivity. However, insulin-based measures are less scalable in real-world screening because insulin assays vary across laboratories and universally transferable cutoffs are lacking across populations with different adiposity and metabolic backgrounds.

#### 4.2.2 Non-insulin-dependent markers

Non–insulin-based indices leverage the fact that dyslipidemia and hyperglycemia are core manifestations of IR. The triglyceride–glucose (TyG) index and composite scores such as METS-IR can be computed from routine clinical data (Table 3), making them attractive for population stratification. Multiple studies across young adults, community cohorts, and pediatric populations have reported positive associations between these indices and prevalent or incident IDH (85–88), with representative effect estimates summarized in Table 4. Notably, effect sizes vary across cohorts, which is expected given heterogeneity in IDH definitions (ACC/AHA vs. ESC/ESH), study settings, and developmental/lifestyle modifiers; sex-specific patterns have also been reported in some datasets (88) (Table 4). Therefore, these indices are best interpreted as scalable tools for metabolic enrichment/risk stratification rather than standalone diagnostic tests for IDH.

Composite indices incorporating anthropometric measures (e.g., TyG-BMI or TyG–waist circumference) may further improve risk signal in some populations (89–91) (Table 3). However, transferability of fixed thresholds is constrained by ethnicity-related body composition differences and measurement variability, so these indices are generally more suitable for within-cohort ranking/stratification than universal cutoff-based diagnosis.Representative population studies linking IR surrogates to IDH prevalence/incidence are summarized in Table 4 (85–88).

#### 4.2.3 Clinical integration

The converging association between IR indices and IDH supports considering these markers in routine cardiovascular assessment, particularly in young adults in whom IDH is prevalent yet often overlooked. A pragmatic approach is to (i) confirm persistent IDH using out-of-office blood pressure monitoring when feasible, (ii) apply non–insulin-based IR surrogates (e.g., TyG, TG/HDL-C, METS-IR) for metabolic enrichment and risk stratification, and (iii) integrate results with obesity/nonalcoholic fatty liver disease (NAFLD) status, family history, and lifestyle factors to reduce misclassification (Tables 1, 3, 4). This logic is operationalized in the stepwise workflow shown in Figure 3, which links phenotype confirmation and IR-informed stratification to tailored management and follow-up.

### 4.3 Impact of metabolic abnormalities on IDH prognosis

The prognostic implications of IR and related metabolic dysregulation in IDH have become an increasing focus. As the central node of the IR–SVR–IDH axis, IR can drive downstream abnormalities—including hyperglycemia, proatherogenic dyslipidemia, chronic inflammation, and procoagulant states—that may accelerate progression from IDH to more advanced hypertensive phenotypes and target organ damage (86, 92). Clinical data are broadly consistent with this framework: higher IR burden has been linked to higher risks of major adverse cardiovascular events in hypertensive populations (92), and TyG has been associated with all-cause and cardiovascular mortality in hypertensive cohorts (93). Importantly, emerging evidence suggests that metabolic status may modify the risk signal of IDH. In a nationally representative NHANES analysis with mortality follow-up, IDH was not associated with cardiovascular or all-cause mortality in participants without diabetes, whereas IDH was associated with significantly higher cardiovascular and all-cause mortality among those with diabetes, with statistically significant interaction by diabetes status (94). These findings support IR/diabetes-informed risk stratification rather than relying on DBP elevation alone. Analyses emphasizing BP subtypes also suggest that TyG may predict future chronic kidney disease across subtypes, including IDH (95), and large prospective cohorts indicate that METS-IR is associated with composite cardiovascular endpoints and mortality among hypertensive patients (96). Together, these observations imply that coupling blood pressure phenotype with metabolic phenotype may better identify high-risk, metabolism-driven subgroups.

At the same time, most outcome evidence comes from cohort studies, frequently as stratified analyses within broader hypertensive populations, with variable follow-up duration and inconsistent ABPM confirmation of persistent IDH (16, 93, 95, 96). These limitations highlight the need for rigorously phenotyped longitudinal studies and randomized trials enrolling ABPM-confirmed persistent IDH and prespecifying IR-based enrichment/stratification to test whether improving IR reduces hard endpoints (Figure 4). Nevertheless, the IR–SVR–IDH framework provides a pathophysiological rationale for incorporating metabolic assessment into prognostic evaluation and moving toward driver-based risk stratification in IDH (28).

## 5 Therapeutic strategies and future directions

The IR–SVR–IDH axis framework has practical therapeutic implications: if a substantial proportion of IDH reflects a DBP-dominant phenotype with increased resistance-vessel load and frequent metabolic comorbidity, management should address upstream IR-related drivers in parallel with blood pressure control. In this section, we summarize lifestyle and pharmacological strategies in a phenotype-oriented manner consistent with the stepwise workflow in Figure 3, and we highlight research priorities aligned with the trial blueprint in Figure 4 (with key phenotyping considerations in Table 1 and pragmatic IR surrogates summarized in Table 3).

### 5.1 Lifestyle interventions targeting IR and elevated SVR

Lifestyle modification is the cornerstone of managing IR-related IDH because it can act on multiple nodes along the IR–SVR–IDH axis. Community-based trials in children and adolescents have shown that programs combining nutritional counseling with increased physical activity can reduce abdominal adiposity (e.g., waist circumference) and improve blood pressure outcomes/reduce the incidence of high blood pressure (97–99). Weight loss achieved through caloric restriction improves fasting glucose, insulin levels, and indices of IR and is accompanied by reductions in DBP (100). Regular physical activity can mitigate mitochondrial dysfunction, oxidative stress, and inflammation (101); longitudinal evidence suggests that exercise-induced improvements in insulin sensitivity may attenuate arterial stiffness (102), and combined aerobic and resistance training has been shown to reduce muscle sympathetic nerve activity in high-risk populations (103, 104). Overall, lifestyle interventions plausibly improve DBP by simultaneously improving IR, autonomic tone, vascular function, and volume-related physiology.

From a dietary perspective, strategies with well-established blood pressure–lowering effects include the DASH diet and sodium restriction. Controlled trials indicate that DASH reliably lowers both SBP and DBP, whereas effects on insulin sensitivity (e.g., HOMA-IR) are more variable (105). A randomized trial published in 2024 in patients with stage 1 primary hypertension found that DASH combined with time-restricted eating produced greater blood pressure reduction than DASH alone, accompanied by decreased extracellular fluid and increased urinary sodium excretion (106). In a feeding cross-over trial in adults with type 2 diabetes (T2D), lowering sodium intake on top of a DASH-like dietary pattern likewise yielded an additional decrease in DBP, suggesting that sodium/volume contributes to blood pressure lowering in a partially independent manner (107). Evidence and boundary: most lifestyle trials were conducted in broader hypertension and/or metabolic-risk populations rather than ABPM-confirmed IDH cohorts, so translation to persistent IDH should be made with attention to phenotyping (Table 1) (105–107).

### 5.2 Pharmacotherapy: balancing antihypertensive efficacy and metabolic consequences

When lifestyle modification is insufficient, pharmacological therapy may be required. Within the IR–SVR–IDH axis framework, drug choice should consider both blood pressure lowering and metabolic consequences (avoiding worsening IR or glycemic control), while prioritizing regimens that reduce resistance-vessel tone and systemic vascular resistance. The 2024 ESC hypertension guidelines emphasize tailoring treatment to overall cardiovascular risk and comorbidities and highlight out-of-office blood pressure monitoring to improve diagnostic and management accuracy (108) (Figure 3, Table 1).

#### 5.2.1 RAAS blockade as a metabolically friendly backbone

ACE inhibitors (ACEIs) and angiotensin receptor blockers (ARBs) are generally preferred options given their efficacy and relatively favorable metabolic safety profile (109). RAAS activation may promote insulin resistance (IR) through oxidative stress, inflammation, and vascular remodeling; conversely, RAAS blockade may improve endothelial function and the metabolic milieu. Telmisartan has partial PPARγ agonist activity and has been reported to improve insulin sensitivity in selected populations (110). In addition, beyond BP lowering *per se*, evidence from randomized trials, comparative studies, and evidence syntheses suggests that some newer ARBs—particularly telmisartan and, in certain settings, olmesartan-based regimens—may be associated with modest improvements in metabolic phenotypes relevant to this framework, including glycemic traits/insulin sensitivity surrogates and lipid profiles (e.g., triglycerides) (111–114). Angiotensin receptor–neprilysin inhibitors (ARNIs) may confer metabolic and hemodynamic benefits through cyclic guanosine monophosphate (cGMP)-related pathways, although direct evidence in IDH remains limited (115). Evidence and boundary: RAAS blockade is guideline-concordant for hypertension, but IR-specific benefits are often based on intermediate endpoints and agent-/population-specific signals, and RCTs in ABPM-confirmed persistent IDH remain lacking (108–115).

#### 5.2.2 Calcium channel blockers to directly reduce resistance-vessel tone

Dihydropyridine calcium-channel blockers (CCBs) reduce small-artery tone and SVR by inhibiting L-type calcium channels, making them suitable as first-line agents or in combination with RAAS blockade; their association with incident diabetes risk appears largely neutral (109). Evidence and boundary: CCBs align well with a high-SVR phenotype but do not directly address IR; therefore, they should be implemented alongside lifestyle and metabolic management in metabolically burdened patients (Figure 3) (109).

#### 5.2.3 β-Blockers, thiazide diuretics, and MRAs: use by indication with explicit metabolic trade-offs

Conventional β-blockers and higher-dose thiazides are associated with less favorable metabolic profiles; vasodilating β-blockers (e.g., carvedilol, nebivolol) appear more metabolically favorable (116–118), and mineralocorticoid receptor antagonists (MRAs) may influence both hemodynamic and metabolic arms but require monitoring for hyperkalemia (108). Evidence and boundary: subtype-specific evidence in IDH remains limited, and metabolic effects depend on dose and baseline IR severity; these agents are best deployed by indication with dose discipline and metabolic surveillance (116–120).

#### 5.2.4 Insulin sensitizers and cardio–renal–metabolic drugs as adjuncts when metabolic indications exist

Metformin improves insulin sensitivity via AMPK activation and exerts beneficial effects on lipid metabolism, inflammation, and endothelial function (119, 121). In populations with T2D and hypertension, SGLT2 inhibitors reduce 24-h ambulatory blood pressure (including DBP), and evidence synthesized from ABPM RCTs and meta-analyses supports small but consistent reductions in 24-h SBP/DBP (122). Glucagon-like peptide-1 receptor agonists (GLP-1RAs) can produce modest DBP reductions (123), and recent RCT meta-analyses suggest benefits in cardiovascular and renal outcomes (124). Evidence and boundary: these trials rarely enroll IDH as the primary inclusion phenotype, and DBP/SVR effects may be partly mediated by weight, volume, and neurohumoral changes; therefore, these agents are best considered when metabolic indications (prediabetes/T2D/obesity) are explicit, rather than as routine substitutes for standard antihypertensive therapy in isolated IDH (122–125) (Figure 3).

### 5.3 Emerging targets and precision-medicine approaches

For emerging targets, it is more productive to prioritize directions that can be translated into testable clinical questions rather than to enumerate many candidate molecules. Representative avenues include key metabolic enzymes/transcriptional regulators (e.g., NNMT), immune–oxidative stress networks, and gut microbiota–derived metabolites; however, the overall evidence base remains predominantly preclinical or mechanistic, and routine clinical translation is still distant (126–133). Rather than asserting that a specific target can treat IDH, a near-term translational strategy is implementable precision stratification: combining ABPM phenotypes (persistent IDH, nocturnal DBP burden, circadian patterns) with pragmatic IR surrogate markers (e.g., TyG, METS-IR, HOMA-IR; Table 3) to enrich for metabolically driven, high-SVR subgroups and then testing within these subgroups whether lifestyle intervention, metabolic medications, or metabolically favorable antihypertensive regimens deliver clearer benefits (134, 135) (Figure 4). Exploratory studies using metabolomics and machine learning provide methodological references for this phenotype-to-intervention pathway (136, 137). Evidence and boundary: multi-omics/AI evidence is mostly associative/predictive and lacks cross-cohort reproducible thresholds and interpretable models, so strict phenotyping, standardized sampling, and prospective validation are required before actionable tools can be established (134–138).

### 5.4 Research priorities and considerations for clinical trial design

Translating mechanistic insights into clinical benefit requires trials designed specifically for IR-related IDH (139) (Figure 4). For enrollment and phenotyping, standardized office blood pressure plus ABPM is recommended to identify persistent IDH and quantify DBP burden, with harmonized ABPM protocols to reduce misclassification (140–142) (Table 1). Recent evidence indicating an independent association between nocturnal DBP and heart failure risk supports including nocturnal DBP and circadian patterns as key phenotypes/endpoints in IDH trials (143). For IR stratification, implementable indices (HOMA-IR or TyG) should be prioritized for enrichment and response monitoring (Table 3), with prespecified subgroup analyses (or continuous-variable modeling) to evaluate dose–response relationships (144–146). In terms of interventions, an integrated metabolic arm + SVR arm approach can be adopted, and factorial designs can test incremental benefits (e.g., lifestyle × metabolic drug, or metabolic drug × antihypertensive regimen) (147). Endpoints should include ABPM-based DBP metrics plus intermediate outcomes reflecting axis progression (arterial stiffness, cardiac structure/diastolic function, metabolic endpoints), alongside adjudicated hard outcomes [e.g., major adverse cardiovascular events (MACE), heart failure hospitalization, chronic kidney disease progression, all-cause mortality] (139, 148). Populations should include a higher proportion of young adults, with prespecified stratification by age, obesity/NAFLD, salt sensitivity phenotype, and nocturnal hypertension/non-dipping patterns to test consistency of benefit (16, 149). Evidence and boundary: given heterogeneity in IDH definitions and natural history—and the fact that IDH is often treated as a subgroup in existing studies—only rigorous phenotyping and long-term follow-up can answer whether targeting IR yields outcome benefits beyond blood pressure reduction itself (16, 139, 149).

## 6 Conclusions

Isolated diastolic hypertension (IDH) is a common yet often overlooked phenotype in young and middle-aged adults (3–5, 21, 22). In many patients, IDH is not a “small-number” variant of hypertension but a resistance-vessel–leaning state characterized by elevated systemic vascular resistance with relatively preserved large-artery compliance (17, 18, 27, 28, 35, 36). Here, we propose an insulin resistance–systemic vascular resistance–IDH (IR–SVR–IDH) axis as a practical metabolic framework for an actionable subset of IDH, while acknowledging substantial etiologic heterogeneity.

Clinically, the first step is to confirm persistent IDH with out-of-office monitoring (ABPM/HBPM) to minimize misclassification (24). For IR-enriched presentations, scalable IR surrogates (e.g., TyG and METS-IR, or insulin-based indices when available) can support early identification and cardiometabolic risk stratification (31, 32, 85–88). Management should prioritize intensive lifestyle intervention and metabolically neutral or favorable antihypertensive regimens that effectively reduce resistance-vessel tone, complemented by cardiometabolic therapies when standard metabolic indications exist (105–109).

Future studies should integrate rigorous BP phenotyping with metabolic stratification to define high-risk IDH subgroups and test driver-informed interventions (94, 140–142).
